# Salivary Concentrations of Chemerin, α-Defensin 1, and TNF-α as Potential Biomarkers in the Early Diagnosis of Colorectal Cancer

**DOI:** 10.3390/metabo12080704

**Published:** 2022-07-28

**Authors:** Dariusz Waniczek, Elżbieta Świętochowska, Mirosław Śnietura, Paweł Kiczmer, Zbigniew Lorenc, Małgorzata Muc-Wierzgoń

**Affiliations:** 1 Department of Surgical Nursing and Propaedeutics of Surgery, Faculty of Health Sciences in Katowice, Medical University of Silesia, 40-055 Katowice, Poland; dwaniczek@sum.edu.pl; 2 Department of Medical and Molecular Biology, School of Medicine with the Division of Dentistry, Medical University of Silesia in Katowice, 40-055 Katowice, Poland; eswietochowska@sum.edu.pl (E.Ś.); pkiczmer@wp.eu (P.K.); 3 Department of Pathomorphology, Faculty of Medical Sciences in Katowice, Medical University of Silesia, 40-055 Katowice, Poland; msnietura@sum.edu.pl; 4 Department of General, Colorectal and Multiple-Organ Surgery, Medical University of Silesia in Katowice, 40-055 Katowice, Poland; zlorenc@sum.edu.pl; 5 Małgorzata Muc-Wierzgoń, Department of Preventive Medicine, Medical University of Silesia in Katowice, 40-055 Katowice, Poland

**Keywords:** colorectal cancer, chemerin, α-defensin 1, TNF alfa, saliva, serum, biomarkers

## Abstract

Colorectal cancer is one of the most prevalent cancers worldwide. There is a great interest and need to find simple, inexpensive, and minimally invasive diagnostic tests. The aim of the study was to analyze the salivary concentrations of chemerin, α-defensin 1, and TNF-α in colorectal cancer (CRC) patients and in a healthy control group. The concentration of these proteins was simultaneously determined in the serum of subjects. We also aimed to assess the correlation of these results and selected clinicopathological features. This prospective study was comprised of 39 CRC patients and 40 control group patients. Salivary and serum concentrations were determined by enzyme immunoassays. The salivary and serum concentrations of chemerin, α-defensin 1, and TNF-α were significantly higher in cancer patients compared to the control group. No correlation was found between concentrations of the proteins and the clinical stage of cancer and tumor location. The ROC curve analysis showed that although salivary concentrations of all proteins showed 100% sensitivity and 100% specificity, serum concentrations of the analyzed proteins were characterized by 100% sensitivity and over 90% specificity. The assessment of chemerin, α-defensin 1, and TNF-α concentrations in saliva seem to have great potential as quick and useful biomarkers in the early diagnosis of CRC.

## 1. Introduction

Colorectal cancer (CRC) is the most common malignant neoplasm of the gastrointestinal tract, and the third most prevalent malignancy in women and men [1]. Carcinogenesis in CRC is the result of combined genetic and environmental factors. The risk of developing the disease increases with age. Other important risk factors for CRC include smoking, obesity, metabolic disease, unhealthy diet, and inflammatory bowel disease (IBD).

There is great interest and need to find simple, inexpensive, and minimally invasive diagnostic tests due to the prevalence and extremely high social costs associated with CRC. It can be achieved by the identification of blood-circulating proteins related to the formation and development of cancer, which allows for early cancer detection and assessment of disease prognosis and prediction. Furthermore, attempts have recently been made to replace routine blood diagnostic methods with the analysis of patient saliva. It seems that in some cases, serum examination can be replaced with the non-invasive collection of saliva, which is easily acceptable to patients.

Chemerin, one of the adipokines which exert multidirectional effects, is mainly produced by adipocytes, liver, and placental cells. Its high expression was also found in the heart, skin, brain, thymus, and pancreas, as well as in the lungs, kidneys, skeletal muscles, and adrenal glands [2,3,4]. The chemerin gene is found on chromosome 7, and chemerin is synthesized as a prepropeptide (preprochemerin), which consists of 163 amino acids converted into inactive prochemerin with a molecular weight of 18 kDa, which is later converted into the active protein with a molecular weight of 16 kDa by proteases [4,5,6]. All chemerin functions have been attributed to activation of the G protein-coupled receptor chemokine-like receptor-1 (CMKLR1) and G protein-coupled receptor-1 (GPR1) [7,8,9]. Chemerin, as a chemoattractant, plays a crucial role in innate and acquired immunity. It exerts mainly proinflammatory effects, and its concentrations are increased in chronic inflammatory diseases such as Crohn’s disease, hepatitis C, pancreatitis, psoriasis, and metabolic disease. The secretion of this hormone is regulated by, e.g., cytokines, mainly tumor necrosis factor α (TNF-α), interleukin (IL)-1β, IL-6 and IL-8, and interferon (IFN)γ [4,10,11,12]. Increased chemerin expression was found in tongue, esophageal, and gastric cancers [3,13,14]. Chemerin exerts effects on tissues through the CMKLR1 receptor, whose activation stimulates angiogenesis, which is of crucial importance for the progression of malignant tumors [15,16].

These observations and associations with obesity, insulin resistance, metabolic disorders [2,17,18], and increased chemerin concentrations in chronic inflammatory diseases, including IBD, suggest that chemerin may play a role in the pathogenesis of CRC, and the increase in its concentration may constitute a potential marker in CRC [2].

Defensins are host defense peptides with a molecular weight of 3–4 kDa, which are highly active against microorganisms in mammals. Human genes encoding defensins are characterized by a high degree of polymorphism, which may be associated with susceptibility to certain diseases, such as Crohn’s disease [19]. Humans produce six α-defensins, which form three intramolecular disulfide bonds between one to six, two to four, and three to five cysteine residues. Human neutrophil peptides (HNPs) 1–4 are four α-defensin peptides expressed in neutrophils, monocytes, lymphocytes, natural killer (NK) cells and dendritic cells. The other two peptides (HD5 and HD6) are expressed in epithelial cells of the gastrointestinal tract, mainly in the Paneth cells [20,21]. α-defensins affect microorganisms directly and indirectly. They have anti-inflammatory and cytotoxic effects by stimulating the synthesis of immune substances such as cytokines, chemokines, and growth factors. At the same time, in humans, α-defensins increase the secretion of TNF-α and IFNγ by neutrophils, which activates and enhances macrophage phagocytosis [22,23]. Increased expression of α-defensins was found in chronically inflamed colon and IBD [23,24]. Several studies also reported increased expression of α-defensins 1–3 in several cancers, including CRC, which suggests their potential use as tumor markers [25,26,27,28,29,30,31].

TNF-α is one of the major inflammatory mediators essential for the proper functioning of the immune system. This pleiotropic cytokine has both pro- and anti-inflammatory effects, pro- and anti-neoplastic activity at various stages of physiopathological processes. It can act as a switch between inflammation and cancer [32,33,34] and is also associated with obesity and insulin resistance [35]. It has an important role in the pathogenesis of different inflammatory conditions, including rheumatoid arthritis and IBD [36,37]. Accumulating evidence shows that TNF-α is associated with all stages of carcinogenesis. TNF-α is a cytokine produced mainly by macrophages and tumor cells [34,35]. Increased expression of TNF-α is found in many neoplasms [36,37,38]. Its increased expression is also reported in the CRC tissue which increases with the cancer stage [39,40,41]. It is also used in the assessment of the prognosis in CRC [39,41].

The aim of the study was to assess salivary and serum concentrations of chemerin, α-defensin 1 and TNF-α in CRC patients and to compare them with the concentrations found in healthy subjects, which could demonstrate that these proteins could be potential diagnostic markers in CRC. We also aimed to assess the correlation of these results with the selected clinicopathological features of the cancer.

## 2. Materials and Methods

### 2.1. Ethics

The current study was conducted in accordance with the Declaration of Helsinki.The project was approved by the Bioethics Committee of the Medical University of Silesia in Katowice-decision No. KNW/0022/KBI/42/14/16/18.

The participants were informed in detail about the study and gave their written consent. Participation in the study was voluntary. Patient data has been encoded in accordance with the pseudonymisation procedure, which means that personal data is processed in such a way that it cannot be assigned to a specific data subject, without the use of an additional “key”.

### 2.2. Patients

This prospective study was comprised of two groups of patients over 60 years of age who underwent surgery in the general surgery department *(n = 79*). The study group included 39 patients (21 women, 18 men; mean age 67.8 ± 10.3) with histologically diagnosed CRC, regardless of the clinical stage. The control group included 40 patients (21 women, 19 men; mean age 64.8 ± 9.4) who underwent surgery due to inguinal hernia or varicose veins of the lower extremities, without any history of cancer.

The exclusion criteria: obesity, smoking, alcohol, and drug abuse, diagnosis of diabetes, chronic liver, kidney diseases and inflammatory bowel disease, including ulcerative colitis and Crohn’s disease and patients with locally advanced rectal cancers requiring neoadjuvant radiochemotherapy.

The research material was collected before cancer surgery. All patients with colon cancer and most patients with rectal cancer were not treated before surgery, except for 4 out of a total of 39 study group individuals who received short-term 5-day neoadjuvant radiotherapy (5 × 5 Gray) and were sampled 7–10 days after first dose (the day before surgery). The relatively low proportion of these patients in the study group and the lack of differences in the mean values of the assessed concentrations between these patients and the rest of the study group seem to confirm the thesis that the applied treatment had no effect on the mean values obtained for the entire study group.

The analysis of the study and control groups was carried out based on the clinical examination and laboratory, imaging, and histological studies. The clinical characteristics of both groups and the comparison of the groups are given in Table S1.

The clinicopathological characteristics of the study group are given in Table 1.

### 2.3. Analytical Methods

Venous blood (5 mL) was aseptically collected from the antecubital vein from each patient. After clotting and centrifugation, serum was stored at −80 °C for further analysis. Salivettes were used to collect saliva. Twenty minutes before its collection, patients refrained from eating and drinking. Then, after rinsing the mouth, the patients intensively chewed the cotton swab for about 3 min. Salivettes were closed and centrifuged according to the standard protocol. The material was stored at −80 °C.

#### 2.3.1. Determination of Chemerin Concentration

Salivary and serum concentrations of chemerin were determined by enzyme immunoassay with the use of the BioVendor LLC test (BioVendor, Brno, Czech Republik, Laboratorini medicina cat. no. RD 191136200R) according to the manufacturer’s instructions. To determine the concentrations of the samples, a calibration curve was prepared by using the standards provided in the kit. Absorbance readings were performed by using the Universal Microplate Spectrophotometer (µQUANT BIO-TEK Inc., Bio-Tek World Headquarters, Winooski, VT, USA) at a wavelength of 450/630 nm. The results were processed with KCJunior software version 1.31.5 (Bio-Tek, Winooski, VT, USA). The sensitivity of the kit was 0.01 ng/mL. The intra- and interassay errors were 5.1% and 8.3%, respectively.

#### 2.3.2. Determination of α-Defensin 1 Concentration

Salivary and serum concentrations of α-defensin 1 were determined by enzyme immunoassay by using the Cloud-Clone Corp test (Houston, TX, USA; cat. no. SEB705Hu) in accordance with the manufacturer’s instructions. To determine the concentrations of the samples, a calibration curve was prepared by using the standards provided in the kit. Absorbance readings were performed by using the Universal Microplate Spectrophotometer (µQUANT BIO-TEK Inc., Bio-Tek World Headquarters, Winooski, VT, USA) at a wavelength of 450 nm. The results were processed with KCJunior software (Bio-Tek, Vinooski, VT, USA). The sensitivity of the kit was 0.137 ng/mL. The intra- and interassay errors were <10% and <12%, respectively.

#### 2.3.3. Determination of TNF-α Concentration

Salivary and serum concentrations of TNF-α were determined by enzyme immunoassay by using the Quantikine immunoassay test (R&D Systems, Minneapolis, MIN, USA; cat. no. DTA00D) according to the manufacturer’s instructions. To determine the concentrations of the samples, a calibration curve was prepared by using the standards provided in the kit. Absorbance readings were performed by using the Universal Microplate Spectrophotometer (µQUANT BIO-TEK Inc., Bio-Tek World Headquarters, California, USA) at a wavelength of 450 nm. The results were processed with KCJunior software (Bio-Tek, Vinooski, VT, USA). The sensitivity of the kit was 6.23 pg/mL. The intra- and interassay errors were 3% and 8.4%, respectively.

### 2.4. Statistical Analysis

The obtained results were statistically analyzed. Distribution of quantitative variables was assessed by using the Shapiro Wilk W-test. Data were presented as mean ± standard deviation and as the median with interquartile range due to non-normal distribution of the examined variables. To compare variables between groups, the Mann–Whitney U-test was used. Associations between variables were assessed by using the Spearman’s rank coefficient. The receiver operating characteristic (ROC) curve analysis was performed to determine the usefulness of the variables as biomarkers of CRC. Statistical analysis was performed by using STATISTICA 13 software version 13.1 (Statsoft Inc., Tulsa, OK, USA). *p* values < 0.05 were considered significant.

## 3. Results

**Figure 1:**
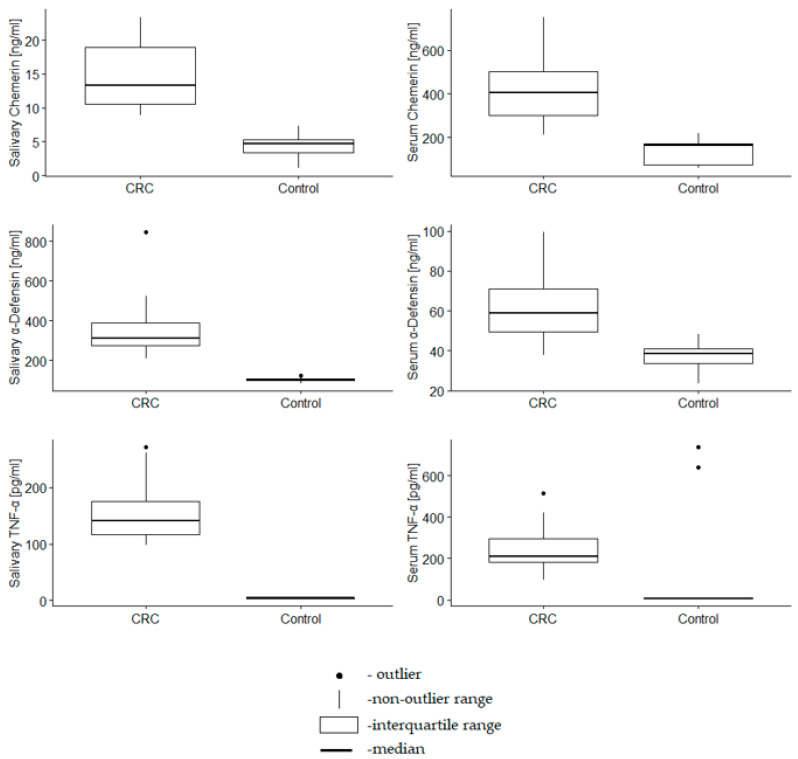
Serum and salivary concentrations of chemerin, α-defensin 1 and TNF-α in CRC patients and control group.

Salivary and serum concentrations of the proteins were significantly higher in CRC patients compared to the control group (*p* < 0.001) (Table 2, Figure 1).

**Figure 2:**
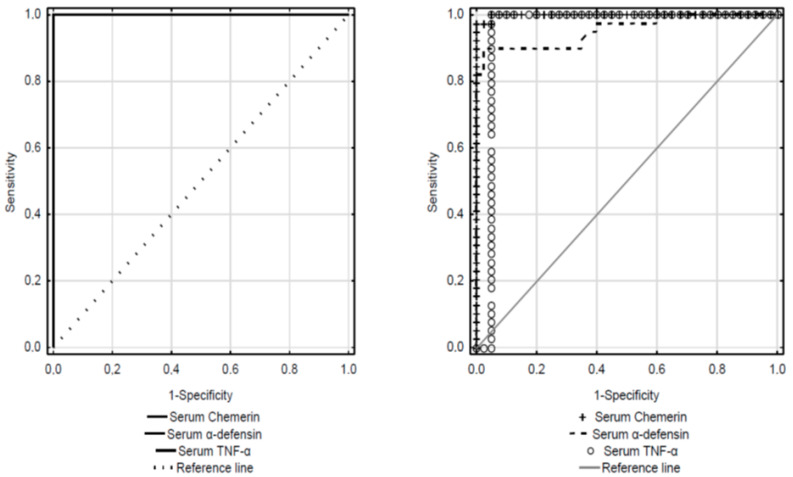
ROC curves for serum and salivary concentrations of chemerin, α-defensin 1 and TNF-α.

To assess the significance of the proteins as markers, a ROC curve was used to calculate the sensitivity of these markers after differentiation between CRC patients and controls. The ROC curve analysis showed that serum concentrations of the proteins were characterized by 100% sensitivity and over 90% specificity, whereas salivary concentrations of all analyzed cytokines showed 100% sensitivity and 100% specificity (Table 3, Figure 2). The cut-off values are also given in Table 3.

Our study did not show a significant influence of the basic anthropometric parameters (i.e., body weight, height, and BMI) on the obtained results. No differences were found in the subgroups of subjects with normal BMI < 25 or overweight/obese subjects with BMI ≥ 25 (Table 4).

The analysis of clinical parameters showed no statistically significant differences between the stages of TNM and of Astler–Coller staging systems or in terms of division into TNM stages I and II or stages III and IV (Table 5).

The increasing trend with the increase in stage was found only in the case of α-defensin 1. No relationship was found between the concentrations of the proteins and tumor location (Table 6).

**Figure 3:**
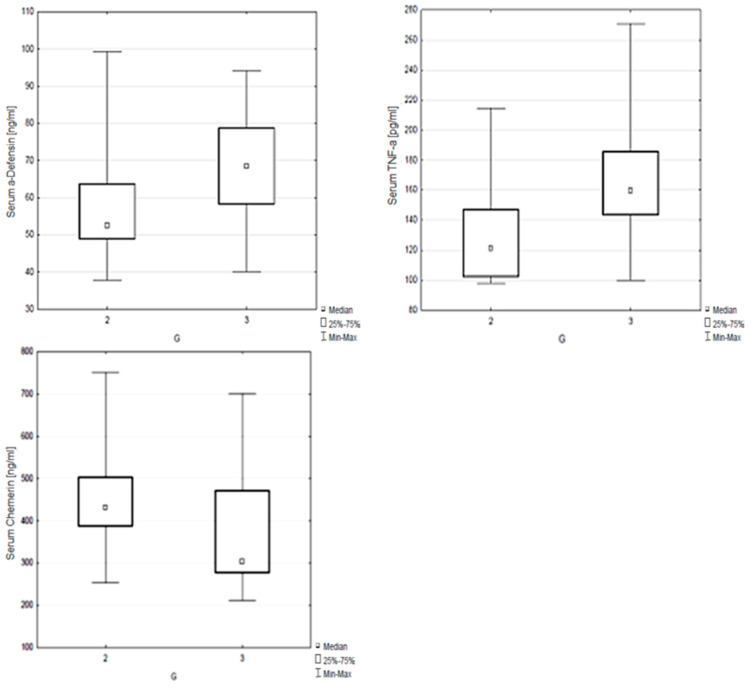
Correlation between serum concentrations of chemerin, α-defensin 1 and salivary concentrations of TNF-α and the tumor grade.

However, we found a clear correlation between serum concentrations of chemerin and α-defensin 1 and salivary concentrations of TNF-α and the tumor grade (*p* < 0.05; Table 7, Figure 3). Patients with G3 grade had lower serum chemerin concentrations and higher serum α-defensin 1 and salivary TNF-α concentrations.

## 4. Discussion

Compared to the control group, high concentrations of the assessed proteins indicated that serum and salivary concentrations of chemerin, α-defensin 1, and TNF-α could be valuable biomarkers in the diagnosis of CRC.

Similar serum results were obtained in a few other studies on chemerin [42,43,44], α-defensins 1–3 [23,24,25], and TNF-α [41,45,46,47]. Furthermore, other studies also showed increased concentrations of these serum markers in other types of cancers [48,49,50,51]. Inflammation is an important risk factor in carcinogenesis. Therefore, increased serum concentrations of chemerin, α-defensin 1, and TNF-α were also reported in many inflammatory diseases, including IBD. However, when IBD and CRC were compared, these values were significantly higher in CRC [8,43,45,52,53]. Additionally, it was suggested that increased concentrations of chemerin and TNF-α were associated with an increased risk of colorectal adenoma, and the associated inflammation was important in the early stage of CRC development [54,55]. However, a meta-analysis of study results showed that TNF-α could not be a useful biomarker for the identification of colorectal adenomas [56].

In our study, we did not confirm that the concentrations of chemerin, α-defensin 1, or TNF-α were significantly different in the advanced CRC stages of TNM. Statistically significant differences were found only in terms of differentiation between the study group with CRC and the control group. An increasing tendency was observed in the case of α-defensin 1. Alkady et al. [43] observed a progressive increase in serum chemerin concentration with cancer stage. The highest difference was found between TNM stage I and II (275.2 ± 58.7 ng/mL vs. 365 ± 34.6 ng/mL). Similarly, another study found an increase in chemerin concentrations, but no correlation with the TNM stage was reported (*p* = 0.063) [43]. Eichelmann et al. [45] showed in a study on a large group of patients that higher concentrations of chemerin were associated with a higher risk of CRC (hazard ratio = 1.81; 95% CI, 1.08–3.05; *p* = 0.007). Additionally, the risk of CRC with increased serum concentrations was higher for the colon than for the rectum, and was the highest for the proximal colon.

We did not find any significant differences between the tumor location and the concentrations of the proteins. Albrethsen et al. [27] did not confirm the relationship between α-defensins 1–3 and the Dukes stage, which is in line with our studies. In terms of TNM classification and tumor grade, we showed that the concentrations of α-defensins 1–3 were already increased in the early stages of the tumor and did not increase with the development of cancer.

However, studies on serum TNF-α in patients with CRC found that these concentrations increased with the stage of cancer and higher mortality [41,45,47]. Stanilov et al. [41] showed a twofold increase in serum TNF-α concentrations in CRC patients compared to the control group. The highest TNF-α concentrations were found in stage IV CRC, which is also associated with poor survival as opposed to some studies in which such a relationship was not reported, which according to those researchers and the authors of this study was due to a small group size [47].

In our results no statistically significant differences were found in the clinical parameters. Height, body weight, and BMI had no influence on the results. We did not find the impact of being overweight in the results, which was also related to chemerin, although the medians of the assessed parameters increased in subjects with BMI ≥ 25 (Table 5). Other studies also found significantly higher concentrations of circulating chemerin in CRC when parameters such as age, sex, BMI, waist circumference, and diet were considered [42].

Although chemerin was related not only to carcinogenesis but also to age-dependent metabolic syndrome, obesity, and insulin resistance, and high concentrations of the markers in cancers did not have such a significant influence on the results [43,44]. No relationship was reported between chemerin concentration and smoking in chronic obstructive pulmonary disease (COPD) [56]. Similarly, it seems that smoking, obesity, and diabetes, which only slightly increase serum TNF-α concentration, do not have a significant influence on the results [54,55,57]. TNF-α was also higher in smokers (in behavioral studies and research on Sjogren’s syndrome) and lower in periodontitis.

We obtained similar values for chemerin, i.e., 99% sensitivity and 100% sensitivity with the cut-off value of 231.24 ng/mL, which also resulted from the selection of the control group without inflammatory diseases and without a history of cancer. Our results showed 100% and 91% specificity for α-defensin 1 and 100% and 95% specificity for TNF-α. Of note, 100% sensitivity and 100% specificity may result from the study limitations. In the future, we plan to expand the study group, which will allow for more accurate assessment of the real usefulness of the analyzed proteins.

Serum and saliva are the most commonly analyzed body fluids, which can contain useful biomarkers for cancer detection. As a clinical tool, saliva has many advantages compared to serum and tissue assessment. It is easy to collect, store, and transport. Additionally, it is safer for medical staff compared to blood and other body fluids. In oncology, an attempt is made to use saliva to determine the concentrations of markers of certain neoplasms such as CRC, oral cavity, gastric, breast, and ovarian cancers [57,58,59,60].

Recently, examination of saliva, which is secreted by multiple large and small salivary glands in the oral cavity, has increasingly replaced routine diagnostic methods. This is supported by many advantages, e.g., easy and multiple collection of the material for examination, even several times a day. Of note, it can be used for screening large populations. Under certain conditions, this non-invasive alternative to serum can replace serum testing in patients with contraindications to phlebotomy or in those in whom blood collection poses some difficulty. Unlike serum examination, assessment of saliva is also associated with lower costs of assessment and the possibility of frequent, repeated testing which is acceptable to patients. Studies should consider age, gender-related differences, and the circadian rhythm of saliva secretion [61,62].

## 5. Limitation

Due to fact that, we recruited a homogenous group of patients and controls, our first study limitation is the fact that we have a limited (39 patients) number of investigated individuals.

Despite the relatively small size of the studied groups, the obtained differences in biomarker concentrations show high statistical significance, which may be an indirect confirmation of the strength of the observed effects. However, in the future, we plan to expand the study group with more patients

The study shows preliminary findings, which we plan to use in the development of a larger research project.

Secondly, our patients from both groups (study and control) most often cited arterial hypertension as a comorbid disease. We know that the chemerin protein levels are elevated in hypertensive patients because experimental and clinical studies support a causative role of chemerin in blood pressure control. But the hypertension is the typical diseases of Europeans over 50 years of age. In the last 30 years, the prevalence of hypertension has decreased to one-fourth of the global population.

Additionally, our results showed 100% and 91% specificity for α-defensin 1 and 100% and 95% specificity for TNF-α. Of note, 100% sensitivity and 100% specificity may result from the study limitations, although we do not expect potential differences in the values of these analytical parameters to be of significant importance for the practical applications of the studied biomarkers. In the future, we plan to expand the study group, which will allow for more accurate assessment of sensitivity and specificity of the analyzed protein levels.

## 6. Conclusions

Our translational research aimed to improve the effectiveness and individualization of treatment of patients. Chemerin, α-defensin 1, and TNF-α seem to be suitable markers in the diagnosis of CRC, particularly in the initial diagnosis of CRC. Therefore, tests based on simple examination of saliva or serum, which measure the concentration of proteins (antigens) associated with carcinogenesis can support and increase the effectiveness of CRC screening. Our results have indicated that further assessment of chemerin, α-defensin 1, and TNF-α concentrations in saliva warrants our continued research on the prospective assessment of these proteins as a simple screening tool for CRC.

## Figures and Tables

**Table 1:** Clinicopathological characteristics of the study group.

Feature	Value
Tumor location	Cecum: 2 (5.13%) Ascending colon: 6 (15.38%) Transverse colon: 3 (7.69%) Descending colon: 1 (2.56%) Sigmoid colon and rectosigmoid junction: 6 (15.36%) Rectum: 13 (33.33%) left colon: 13 (33.33%) Left colon and rectum: 26 (66.67%) Colon: 26 (66.67%) Rectum: 13 (33.33%)
Gender	Male:18 (46.2%); Female: 21(53.8%)
Grading	G1:0 (0%); G2: 22 (56.4%); G3: 17(43.6%)
T	I: 1 (2.56%); II: 16 (41.03%); III: 20 (51.28%); IV: 2 (5.13%)
N	N0: 24 (61.54%); N1: 4 (10.26%); N2: 11 (28.21%)
M	M0: 36 (92.31%); M1: 3 (7.69%)
TNM staging	I: 14 (35.9%); II: 9 (23.08%); III: 13 (33.33%); IV: 3 (7.69%)
Astler-Coller staging	A1: 1 (2.56%); B1: 13 (33.33%); B2: 8 (20.51%); C1: 3 (7.69%); C2: 11 (28.21%); D: 3 (7.69%)

**Table 2:** Serum and salivary concentrations of chemerin, α-defensin 1 and TNF- α (ng/mL) in CRC patients and control group.

Marker Concentration	Control			CRC			*p*
	Median	Q1	Q3	Median	Q1	Q3	
Serum chemerin (ng/mL)	164.07	73.37	173.39	406.52	302.54	502.10	<0.001
Salivary chemerin (ng/mL)	4.71	3.39	5.25	13.28	10.21	18.94	<0.001
Serum α -defensin1 (ng/mL)	38.51	33.58	40.95	58.83	49.47	72.84	<0.001
Salivary α -defensin1 (ng/mL)	104.39	98.23	108.95	310.92	274.09	398.84	<0.001
Serum TNF α (ng/mL)	7.23	6.49	7.87	207.66	176.59	302.31	<0.001
Salivary TNF α (ng/mL)	4.60	3.83	5.26	141.28	115.91	176.48	<0.001

Q1, Q3—quartile; *p*—statistical significance of the Mann–Whitney U test.

**Table 3:** ROC curve analysis for differentiation of patients with CRC and healthy subjects for chemerin, α-defensin 1 and TNF- α in serum and saliva for *p* <0.05.

	Cut-Off Value	AUC	SE	Lower AUC 95%	Upper AUC 95%	z-Score	*p*	Specificity	Sensitivity
Serum chemerin	231.24	1.00	0.00	1.00	1.00	281.31	0.00	1.00	0.99
Salivary chemerin	8.85	1.00	0.00	1.00	1.00	-	0.00	1.00	1.00
Serum α-defensin1	46.79	0.95	0.02	0.91	1.00	19.69	0.00	1.00	0.91
Salivary α-defensin 1	211.46	1.00	0.00	1.00	1.00	-	0.00	1.00	1.00
Serum TNF-α	95.64	0.95	0.03	0.88	1.00	13.06	0.00	1.00	0.95
Salivary TNF-α	97.86	1.00	0.00	1.00	1.00	-	0.00	1.00	1.00

AUC—area under curve, SE—standard error.

**Table 4:** Comparison of serum and salivary concentrations of chemerin, α-defensin 1, and TNF-α in all groups depending on BMI.

Marker Concentration	BMI < 25			BMI ≥ 25			*p*
	Median	Q1	Q3	Median	Q1	Q3	
Serum chemerin (ng/mL)	190.26	163.22	302.54	289.39	164.92	439.01	0.18
Salivary chemerin (ng/mL)	5.64	4.88	11.45	9.49	4.27	14.37	0.82
Serum α-defensin 1(ng/mL)	41.92	36.36	51.91	45.75	39.71	63.84	0.27
Salivary α-defensin 1(ng/mL)	110.52	102.37	299.31	213.04	107.28	313.67	0.40
Serum TNF-α (ng/mL)	9.35	7.23	202.37	175.63	7.23	221.37	0.40
Salivary TNF-α (ng/mL)	6.15	4.57	118.36	99.29	4.62	143.49	0.56

**Table 5:** Concentrations of the markers depending on the stage according to the TNM classification: tumors without metastases (stages I and II) and with distant metastases and/or local lymph node metastases (stages III and IV).

Marker Concentration	Stage I + II			Stage III + IV			*p*
	Median	Q1	Q3	Median	Q1	Q3	
Serum chemerin (ng/mL)	384.69	265.18	491.30	416.09	375.91	502.10	0.24
Salivary chemerin (ng/mL)	16.04	10.36	20.72	12.47	10.21	17.65	0.42
Serum α-defensin 1(ng/mL)	66.22	50.00	81.10	55.22	49.47	65.67	0.25
Salivary α-defensin 1(ng/mL)	322.15	281.92	465.06	285.63	254.20	318.12	0.10
Serum TNF-α (ng/mL)	229.56	196.00	305.51	205.61	153.27	286.01	0.12
Salivary TNF-α (ng/mL)	149.17	114.05	176.81	131.06	115.91	176.48	0.47

Q1, Q3, quartiles; *p*, statistical significance of the Mann–Whitney U test.

**Table 6:** Serum and salivary concentrations of chemerin, α-defensin 1 and TNF-α depending on the tumor location.

Marker Concentration	Rectum			Colon			*p*
	Median	Q1	Q3	Median	Q1	Q3	
Serum chemerin (ng/mL)	389.50	261.45	472.10	411.31	312.84	503.66	0.40
Salivary chemerin (ng/mL)	13.28	10.90	18.82	14.63	10.21	20.07	0.48
Serum α-defensin	55.22	46.79	68.59	61.82	51.00	72.84	0.36
Salivary α-defensin	285.63	227.58	316.25	312.62	277.74	404.20	0.17
Serum TNF-α	206.58	189.63	261.36	212.35	175.63	304.51	0.73
Salivary TNF-α	132.75	124.12	159.74	145.44	109.73	179.84	0.75
	**Left**			**Right**			
Serum chemerin (ng/mL)	427.55	298.16	541.23	386.77	312.84	425.06	0.38
Salivary chemerin (ng/mL)	12.43	10.12	18.82	16.73	11.53	21.08	0.11
Serum α-defensin	58.51	49.47	72.84	61.17	51.00	68.95	0.99
Salivary α-defensin	312.62	236.02	398.84	293.96	277.74	373.47	0.96
Serum TNF-α	207.12	176.59	271.49	216.54	197.68	305.41	0.53
Salivary TNF-α	131.91	116.49	179.84	153.05	103.79	164.62	0.74

Left, left colon; right, right colon and rectum; *p*, statistical significance of the Mann–Whitney U test; Q1, Q3, quartiles.

**Table 7:** Correlation between serum and salivary concentrations of chemerin, α-defensin 1, and TNF-α and the tumor grade (G).

Marker Concentration	G2			G3			*p*
	Median	Q1	Q3	Median	Q1	Q3	
Salivary chemerin (ng/mL)	12.47	11.29	18.92	16.32	10.21	20.36	0.42
Serum chemerin (ng/mL)	439.01	387.95	503.66	303.45	276.51	472.10	**0.04**
Salivary α-defensin (ng/mL)	310.92	275.64	318.12	293.96	274.09	404.20	0.79
Serum α-defensin (ng/mL)	51.91	48.90	62.47	68.59	58.19	78.84	**0.03**
Salivary TNF-α (ng/mL)	124.12	103.79	147.38	159.74	143.49	185.72	**0.01**
Serum TNF-α ng/mL)	205.71	175.63	286.01	221.37	197.68	305.41	0.35

Q1, Q3, quartiles; *p*, statistical significance of the Mann–Whitney U test.

## Data Availability

The data supporting this article are available upon request to the corresponding author. The data are not publicly available due to maintaining a high level of privacy for the study subjects.

## Supplementary Materials

The following supporting information can be downloaded at: https://www.mdpi.com/article/10.3390/metabo12080704/s1,Table S1: Clinical characteristics of the study group and the control group
