# Supplementary material for: Salivary Concentrations of Chemerin, α-Defensin 1, and TNF-α as Potential Biomarkers in the Early Diagnosis of Colorectal Cancer
Source: Metabolites. 2022 Jul 28;12(8):704. doi: 10.3390/metabo12080704 (PMC9416224; doi:10.3390/metabo12080704)
Supplement: Supplementary file 1 [file metabolites-12-00704-s001.zip › metabolites-1772426-supplementary.pdf]

Supplementary Materials:

Table S1. Clinical characteristics of the study group and the control group

**Variable**	**Control group n=40**	**CRC group n=39**	**p**
Age	M=64.00<br>Q1=59.50; Q3=71.00	M=68.00<br>Q1=59.00; Q2=77.00	n.s
Gender	Male: 19 (47.5%)<br>Female: 21 (52.5%)	Male: 18 (46.2%)<br>Female: 21(53.8%)	n.s
Height	M=170.00<br>Q1=164.00; Q2=176.00	M=170.00<br>Q1=163.00; Q2=176.00	n.s
BMI	M=25.31<br>Q1=23.20; Q2=27.60	M=27.17<br>Q1=23.42; Q2=30.04	n.s
Body weight	M=71.00<br>Q1=65.50; Q2=81.50	M=75.00<br>Q1=63.00; Q2=89.00	n.s
Arterial Hypertension	Hypertension: 19 (47.5%)<br>No hypertension: 21 (52.5%)	Hypertension: 14 (35.9%)<br>No hypertension 25 (64.1%)	n.s.

n - number of subjects; M – median; Q1,Q3 - quartile; p-value of the Mann-Whitney U-test, n.s - not significant.
